# Self-image threat decreases stereotyping: The role of motivation toward closure

**DOI:** 10.1007/s11031-016-9582-6

**Published:** 2016-09-24

**Authors:** Małgorzata Kossowska, Marcin Bukowski, Ana Guinote, Piotr Dragon, Arie W. Kruglanski

**Affiliations:** 1 Institute of Psychology, Jagiellonian University, Ingardena 6, 30-060 Kraków, Poland; 2 University College London, London, UK; 3 University of Maryland, College Park, MD USA

**Keywords:** Need for closure, Self-image threat, Stereotypical perception, Self-esteem, Fear of invalidity

## Abstract

Some prior research indicated that self-image threat may lead people to stereotyping and prejudiced evaluations of others. Other studies found that self-image threat may promote less stereotypical thinking and unprejudiced behavior. In a series of three studies, we demonstrate that self-image threat may lead to either more or less stereotypical perception of the outgroup depending on the level of the individuals` motivation toward closure (NFC). The results reveal that when individuals high (vs. low) in NFC perceived a member of an outgroup, they are less likely to use stereotypical traits if their self-image had been threatened by negative feedback (Study 1) or if they had imagined an example of their own immoral activity (Studies 2 and 3). Moreover, our results demonstrate that the fear of invalidity resulting from self-image threat induction is responsible for the foregoing effects (Study 3). These results are discussed in light of theories of motivational readiness and lay epistemics.

## Introduction

Prior studies yielded results suggesting that self-image threat may lead people to engage in stereotypical thinking and making prejudiced evaluations of others (e.g., Abrams and Hogg 1988; Brown and Gallagher 1992; Crocker et al. 1987; Ehrlich 1973; Fein and Spencer 1997; Gibbons and Gerrard 1991; Stephan and Rosenfield 1978). According to this view, derogating others allows individuals to effectively regain their self-worth. Other studies, however, found that self-image threat may not lead to stereotypical thinking and prejudiced behavior, based on the notion that after people misbehave (e.g., act immorally), they regain their self-worth by behaving well or morally (e.g., Brockner and Chen 1996; Monin and Miller 2001; Petersen and Blank 2003; Sachdeva et al. 2009; Seta and Seta 1992). Yet other studies demonstrate that without the consideration of moderators, there appears to be no clear main effect of self-image threat on stereotyping and prejudice towards outgroup targets (see: Brockner and Chen 1996; Florack et al. 2005).

For example, Florack et al. (2005) showed that a consideration of intergroup attitudes and ingroup identification clarify the relation between self-image threat and stereotypical evaluations of the outgroup. Participants were more likely to derogate the outgroup target as a consequence of self-image threat when they held stereotypes and negative attitudes towards the outgroup. However, a more positive perception after a self-image threat occurred for participants who felt less identified with their ingroup and who did not show a strong preference for it over the outgroup. In the present article we focus on another pertinent variable, i.e., need for closure (NFC, Kruglanski 1989), that may moderate the self-image threat and stereotyping relations.

### Self-image threat, need for closure and stereotypical perception

NFC has been shown to induce the feeling of discomfort experienced in the face of cognitive uncertainty (Webster and Kruglanski 1994). Accordingly, research demonstrated that individuals with high NFC often use restricted cues or crude categories, resulting in biased judgments, stereotyping or prejudice (e.g., Driscoll et al. 1991; Shah et al. 1998; Kruglanski et al. 2002). It appears, however, that sometimes closure, and thus uncertainty reduction, is not always achieved via simplified knowledge structures, such as stereotypes or prejudice; occasionally, more complex cognition appears to be needed to achieve certainty (c.f., Roets et al. 2015). Indeed, studies have shown that NFC is associated with stereotypical impression of the outgroup, but only in presence of an initial, satisfactory basis for closure, whether resulting from sufficiently strong confidence in the initial guess. For example, it was shown that high NFC individuals develop stereotypical impressions about the outgroups under self-esteem threat but only when they held explicit prejudiced beliefs towards the outgroup (Kosic et al. 2014). However, if high (vs. low) NFC individuals lack a prejudice towards outgroup, their sample new information in their quest for a clear-cut answer, thus may develop less stereotypical evaluations of the outgroups (see also Kossowska et al. 2015).

We argue that discrepancy between the need to maintain a positive self-image and threatening information about the self may serve as a signal that existing scripts, rules and other knowledge structures no longer provide guidance on how to act, and thus that they no longer afford a sense of predictability (e.g., Guinote et al. 2006). In that situation, a fear of invalidity is induced (Kruglanski 1989; Thompson et al. 2001). This fear concerning the consequences of an erroneous decision, is manifested in a hesitation reflected in longer response latencies and lowered subjective confidence (Freund et al. 1985). Under those circumstances, inconsistent information would be given more equal weight and consideration, leading to vacillation between alternatives.

Due to their intolerance of uncertainty and aversion to ambiguity, individuals high (vs. low) in NFC, are more sensitive to signals that pre-existing knowledge is unreliable and may be inefficient in reducing uncertainty (Kruglanski et al. 2012). Recently, Kruglanski et al. (2014), building on prior relevant conceptions that include, among others, animal learning models (Hull 1943; Spence 1937; Tolman 1955) and personality approaches (e.g., Atkinson 1964; Lewin 1935), argued that major elements of the willingness to act in the service of a need (i.e., motivational readiness), include the magnitude of a *Want* state (i.e., individual’s need of some sort, e.g., NFC) and the *Expectancy* defined as the subjective probability (i.e., confidence) an individual assigns (consciously or unconsciously) to gratification of the *Want*. Thus, the *Expectancy* construct denotes a subjective likelihood of *Want* satisfaction often stemming from the availability of a specific act perceived as instrumental to goal attainment. If *Want* x *Expectancy* defines the individual’s *Goal* to attain closure, reduction of confidence in one`s knowledge should reduce the *Goal* strength more for those whose *Want* to attain closure is high (i.e., high NFC) than for those whose *Want* to attain closure is lower (i.e., low NFC). Accordingly, people high in NFC who are exposed to information that is distinct from, inconsistent with, and even contradictory to their internal self-representations, may experience a reduction in self-confidence and consequently desist from their habitual reliance on pre-existing knowledge structures regarding typical attributes of outgroup members. Instead they may become more sensitive to non-stereotypic information and attend to and examine more extensively individuating information about groups, resulting in more balanced perception of group targets. As explained above, we did not expect low NFC participants to change their social perceptions of outgroups under self-image threat to quite the same extent.

### Overview of the studies

We tested the above assumptions in a series of three studies, by providing negative feedback regarding participants performance in bogus IQ test (Study 1) or leading participants to imagine that they were engaged in an immoral activity (Studies 2 and 3). We then measured participants’ negative attitudes towards different outgroups: homosexuals (Study 1), Gypsies (Study 2) and Jews (Study 3). In Study 3 we directly tested the possibility that self-image threat induced a fear of invalidity assumed to mediate the self-threat/stereotypical perception effect of present interest. Moreover, we checked whether such fear is responsible for differential effects of self-image threat on stereotypical perception of people who are low versus high in NFC. In Study 3, we also aimed at ruling out the alternative explanation that it is low self-esteem, rather than induced fear of invalidity, that is affected by self-image threat, influences stereotypical perception of the outgroup.

## Study 1

In Study 1, we tested the hypothesis that when individuals’ self-image is threatened by negative feedback, high (but not low) NFC individuals will use fewer stereotypes when forming impressions.

## Method

### Participants

Fifty-one university students (33 females and 18 males; *M*_age_ = 22.31, *SD* = 3.32) participated in the study on a voluntary basis. Four of them were excluded from the analysis because they did not answer the NFC scale, thus in the final analysis we included 47 participants. The students were randomly assigned to the control or self-image threat conditions. Before recruiting participants we performed a power analysis^1^.

### Materials and procedure

At the beginning of the session, participants completed the Need for Closure Scale (Webster and Kruglanski 1994). We used four of the five subscales of this scale: preference for order and structure in the environment; predictability of future contexts; affective discomfort occasioned by ambiguity; and closed-mindedness. We excluded the decisiveness^2^ subscale because it was recognized to measure ability, rather than motivation (Roets and Van Hiel 2007). The respondents rated 27 items on a scale from 1 (completely disagree) to 6 (completely agree). A higher mean score indicated a higher NFC (Cronbach’s *α* = .75, *M* = 4.07; *SD* = 0.65).

To induce a self-image threat we followed the procedure^3^ used by Fein and Spencer (1997). Participants were told that they would be given a series of verbal intelligence tasks that involved analogies, syllogisms and logical reasoning problems (based on the Quick Test of Intelligence; Ammons and Ammons 1962). After the study phase, participants were presented with the IQ test feedback. Half the participants were randomly assigned to the negative feedback (self-image threat) condition, and the other half—to the neutral feedback (control) condition. In the negative feedback condition participants received the following evaluation: “Your score is 7 points, which means that it is low and your rank falls within the lower 30 % of your age group.” In the neutral feedback condition participants received instead the evaluation: “Your score is 7 points. However, since this test is not yet a well established tool, there are no norms for your population.”

After administering a series of brief cognitive tasks designed to enhance the integrity of the cover story, the experimenter introduced the “social judgment tasks” by informing participants that they would be presented with some information about an individual named Greg and then be asked to make a number of judgments about him. In the story, Greg was depicted as a homosexual, i.e., he was said to be in a relationship with a male partner.

Participants used a 10-point scale ranging from 0 (not at all) to 10 (extremely) to rate Greg’s personality on each of 10 dimensions. Three of these (intelligent, funny, and boring) were included as stereotype-irrelevant fillers. The stereotype-relevant traits included assertive/aggressive, considerate, strong, and passive (see Fein and Spencer 1997). Assertive/aggressive and strong were reverse-coded so that for each item, higher ratings indicated greater stereotyping. An index of this set of traits showed moderate internal reliability (Cronbach’s *α* = .66). It may be worth noting that these traits, when taken out of a stereotyped context, are not necessarily negative. Fein and Spencer (1997) demonstrated however that participants perceived these traits as more descriptive of a target if they thought that the target was homosexual than if they thought he was heterosexual.

## Results and discussion

We analyse the data using PROCESS macro (Hayes 2013, model 1). The experimental conditions were coded (0 control/1 self-image threat). As predictor we used experimental condition, as a moderator NFC, and stereotype index as a dependent variable. NFC was mean centered prior to analysis. We also controlled for gender (it was added as a covariate). Additionally, as recommended by Hayes and Cai (2007), we used heteroscedasticity-consistent standard errors estimator.

**Fig. 1:**
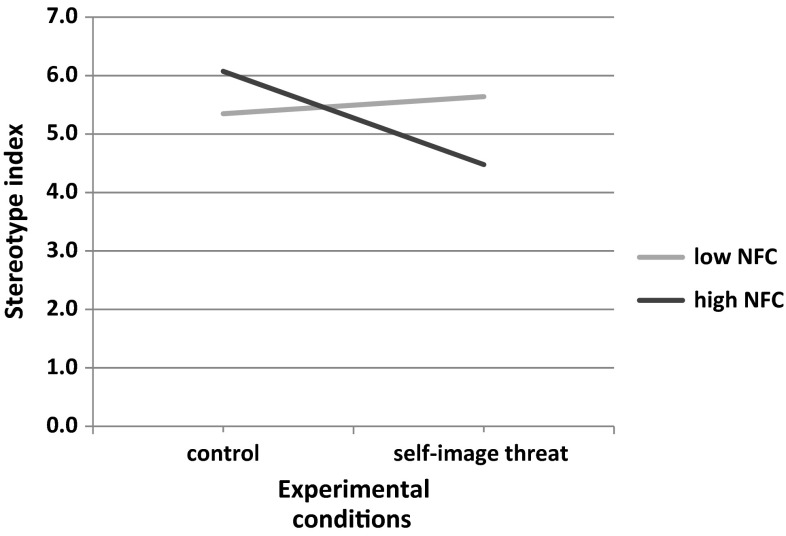
Regression lines showing stereotypical use of information as a function of self-image threat and NFC (Study 1)

We found the marginal main effect of the self-image threat (vs. control) condition (*b* = −.65*; t*(42) = 1.91; *p* = .064; 95 % CI [−1.34, 0.04]), showing that participants in the self-image threat condition stereotyped less than those in the control condition (but as can be seen by examining the pattern of interaction, this effect was solely due to high NFC participants). There was no main effect of gender (*b* = .41*; t*(42) = 1.19; *p* = .243; 95 % CI [−0.28, 1.10]). The only other statistically significant effect was the interaction term between condition and NFC (*b* = −1.45*; t*(42) = 2.75; *p* = .009; 95 % CI [−2.51, −0.39]). The interaction pattern is depicted in Fig. 1.

To probe for the significant interaction, we used a simple slope analysis and calculated the effect of our predictor on DV at low (−1 SD) and high (+1 SD) values of the moderator (NFC). Analysis indicated that self-image threat was negatively related to the stereotype index for people high in NFC (*b* = −1.60; *t*(42) = 3.01*; p* = .004; 95 % CI [−2.66, −.53]) but was not related to this index for people low in NFC (*b* = .29*; t*(42) = .67; *p* = .509; 95 % CI [−0.59, 1.17]).

Thus, in Study 1, we demonstrated that high NFC is associated with less stereotyping in self-image threat condition than in control condition. There were no differences at low NFC levels. It appears that under self-image undermining circumstances, people who score high on NFC rely less on preferred processing styles and do not effectively apply dominant cognitive schemas, such as stereotypes. Specifically, self-image threat manipulation is hypothesized to induce a fear of invalidity which may reduce one’s initial confidence in one’s self-knowledge. That reduction should be proportionately greater for high NFC individuals than for low NFC individuals. This supposition is in line with motivational readiness theory (Kruglanski et al. 2014) suggesting that reduction of confidence in one`s knowledge reduces *Goal* strength more for those whose *Want* to attain closure was higher (i.e., high NFC) than for those whose *Want* to attain closure was lower (i.e. low NFC).

## Study 2

In Study 2, we aimed to replicate the results of Study 1 using a different self-image threat manipulation. We focused on immoral behaviors as an example of self-threatening behaviors, as morality is widely considered to be one of the defining characteristics of a person and has an important impact on cognitive and social functioning (e.g., Bliss-Moreau et al. 2008; Cosmides 1989; Cottrell et al. 2007; Wojciszke 2005). We posited that when high (vs. low) NFC individuals would find themselves displaying immoral behavior, they would perceive outgroup`s members in less stereotypical manner.

## Method

### Participants

Fifty-six students in a secondary school (42 females and 14 males; *M*_age_ = 17.39, *SD* = 1.24) participated in the study as volunteers during a class activity. The participants were randomly assigned to one of two conditions: control and self-image threat.

### Materials and procedure

At the beginning of the session, participants completed the Need for Closure Scale (Webster and Kruglanski 1994). As in Study 1, we used four of the five subscales of this scale (Cronbach’s *α* = 0.74, *M* = 4.08; *SD* = 0.81).

To induce the self-image threat manipulation, we asked participants to imagine that they cheated during an important exam and were caught by the teacher, consequently failing the exam. We expected that for some students, cheating during an exam would not be perceived as immoral behavior (see Węglarczyk 2001). Therefore, to make the manipulation stronger, we added that the behavior is socially exposed and they are punished for it. In the control condition, participants were asked to imagine that they took an important exam. In both conditions, participants were asked to write what they thought and felt.

The experimenter then introduced a second, ostensibly unrelated study. According to the instructions, this study investigated the way people think about different national groups. The participants were asked to assess how Gypsies are considered in Polish society. Participants were told that we were not interested in the participants’ personal beliefs, but rather in how they thought Gypsies were viewed by others. This instruction was recommended by Cuddy and colleagues (Cuddy et al. 2008) because it elicits cultural beliefs and minimizes social desirability. Following the description, participants rated typical Gypsies on 13 stereotypical characteristics (unreliable, educated, lazy, friendly, competent, moral, dishonest, family man, orderly, neat, intrusive, insolent, filthy), tested in a previous study by Kofta and Narkiewicz-Jodko (2003). Specifically, participants assessed on a 7 point scale to what extent they agreed that typical Gypsies possessed these characteristics (Cronbach’s α = .77; *M* = 3.46; *SD* = 1.54). Educated, friendly, competent, moral, and orderly characteristics were reversed before calculating the index of stereotypes. Thus almost all positive characteristics were reverse-coded, but it was because the stereotypical perception of Gypsies in Poland is highly negative—contemptuous stereotype in Stereotype Content Model terminology (Cuddy et al. 2008; Cichocka et al. 2015). The exception here is “family man”, as stereotypical but positive characteristic of Gypsies. The higher the index, the more stereotypical the perception of the outgroup. Participants were subsequently debriefed and thanked.

## Results and discussion

We analysed the data in the same way as in Study 1, using PROCESS macro (Hayes 2013, model 1). The experimental conditions were coded (0 control/1 self-image threat). As before, as predictor we used experimental conditions, as a moderator NFC, and stereotype index as a dependent variable. NFC was mean centred prior to analysis. We controlled for gender. We also applied heteroscedasticity-consistent standard errors estimator.

**Fig. 2:**
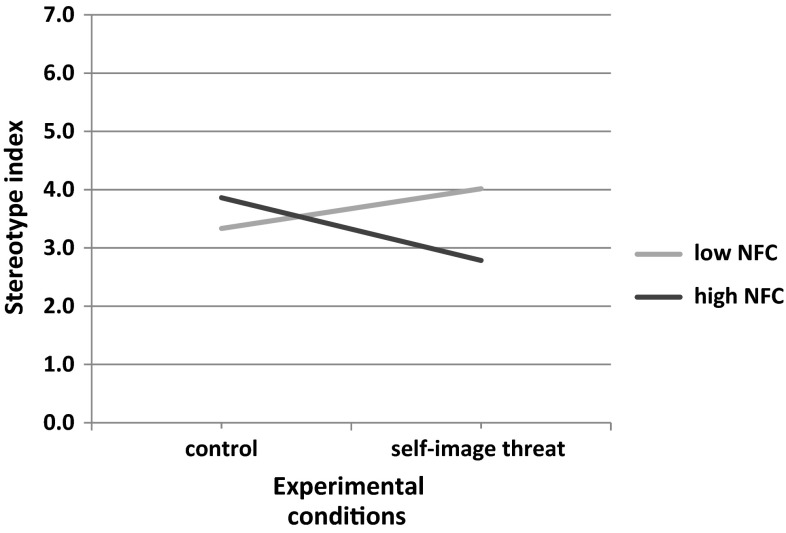
Regression lines showing stereotypical use of information as a function of self-image threat and NFC (Study 2)

There was no main effect of self-image threat (vs. control) condition on the stereotype index (*b* = −.20; *t*(51) = 0.47*; p* = .644; 95 % CI [−1.05, 0.65]). This time we found significant effect of gender (*b* = −1.15*; t*(51) = 3.06; *p* = .004; 95 % CI [−1.91, −0.40]), indicating that women (dummy coded as 1) stereotyped less than men. The only other statistically significant effect was the interaction term between condition and NFC (*b* = −2.06*; t*(51) = 2.01; *p* = .050; 95 % CI [−4.13, 0.00]). The interaction pattern is depicted in Fig. 2.

To probe for the significant interaction, we used a simple slope analysis and calculated the effect of our predictor on DV at low (−1 SD) and high (+1 SD) values of the moderator.

The analysis indicated that self-image threat was negatively related to the stereotype index at high NFC levels (*b* = −1.08*; t*(51) = 2.03*; p* = .048; 95 % CI [−2.14; −0.01]) but it was not related at low NFC levels (*b* = .68*; t*(51) = 1 .00; *p* = .321; 95 % CI [−0.68; 2.05]).

Thus, in Study 2, we demonstrated that self-image threat leads to less stereotypical perception among people high in NFC—as compared to control condition—but not among those who are low in NFC. This result replicated the findings obtained in Study 1. Instead applying stereotypes, people high in NFC attend to and examine more, also non-stereotypical information. In result, they develop a more balanced perception of the outgroup.

In this study we induced self-image threat by asking participants to imagine a situation where they behaved immorally. This manipulation yielded results analogous to those obtained in the previous study, thus further confirming the joint moderating role of self-image threat and NFC on stereotypical perception of the outgroup. Although, we claimed that the mechanism responsible for these findings is induced fear of invalidity, we didn`t measure it directly in either Study 1 or Study 2. Thus in the next study we set out to investigate whether this particular fear mediates the abovementioned effects of self-image threat on stereotypical evaluations at high (vs. low) NFC levels. We also attempted here to rule-out the possibility that self-esteem is responsible for these effects.

## Study 3

In Study 3, we aimed to demonstrate that self-image threat induces a fear of invalidity and that this fear is responsible for less stereotypical perception at high (vs. low) levels of NFC. In addition, to ascertain that the previous effects were driven by fear of invalidity and not by a confounding variable, we examined the role of self-esteem. Fein and Spencer (1997) observed that self-image threat can cause people to explicitly stereotype and derogate outgroup members as a means of restoring their self-esteem. Thus, in Study 3, we intended to demonstrate that although self-image threat manipulation may decrease self-esteem, lower levels of self-esteem are not responsible for the above mentioned effects.

## Method

### Participants

A total of 74 secondary school students (32 females and 42 males; *M*_age_ = 17.26, *SD* = .50) participated in the study on a voluntary basis. The students were randomly assigned to the control or self-image threat condition.

### Materials and procedure

As in Study 1 and 2, we used the NFC scale (Webster and Kruglanski 1994) (excluding the decisiveness subscale; Cronbach’s *α* = .80, *M* = 3.75; *SD* = 0.60). A higher mean score indicated a higher NFC. To induce the self-image threat, we used the same manipulation as in Study 2. To verify whether self-image manipulation induced a fear of invalidity, participants completed the four-items scale^4^ of Thompson et al. (1986). The scale items were as follows: “I hesitate to make important decisions, even after long deliberation” or “I often feel stressed when I have to make an unequivocal decision” (high fear of invalidity), “I make even important decisions quickly and confidently”, “I do not bother with simple matters, usually I know what to do at once” (low fear of invalidity, items were reversed). Participants rated these items on a scale ranging from 1 (not at all) to 6 (very much). We calculated the overall mean score of all these items (Cronbach’s α = .75, *M* = 3.92, *SD* = 0.75). A higher score on the scale implied a higher fear of invalidity.

Next, participants completed 3 items from Heatherton and Polivy’s (1991) State Self-Esteem Scale (e.g. “I feel good about myself”) (Cronbach’s α = .81; *M* = 3.78; *SD* = 0.69). Participants indicated how true the statements were for them “right now,” ranging from 1 (not at all) to 5 (extremely).

The experimenter then introduced a second, ostensibly unrelated study. We used a similar stereotypical perception measure as in Study 2. Specifically, following the description, participants evaluated a typical Jew on 12 traits using a seven-point Likert scale, from 1 (not at all) to 7 (extremely). Eight of the traits were stereotypical (intelligent, competent, competitive, independent, insincere, bad-natured, cold, and intolerant; these traits constitute envious stereotype, Cuddy et al. 2008), and the remaining four were filler traits (practical, optimistic, family men, joyful). These stereotypical traits were tested in a previous study by Kofta and Sedek (2005). From assessments of the stereotypical traits, we calculated the mean score for stereotypical perception of typical Jew and as in previous studies labelled it the stereotype index, such that the higher the index, the more stereotypical perception (Cronbach’s α = .79). Participants were subsequently debriefed and thanked.

## Results and discussion

### Manipulation checks

To investigate whether self-image threat manipulation affected the fear of invalidity, an independent *t* test (control vs. self-image threat) was conducted on the scale. As expected, participants in the self-image threat condition reported a higher fear of invalidity (*M* = 4.08; *SD* = 0.82) than participants in the control condition (*M* = 2.10; *SD* = 0.97), *t*(72) = 9.46, *p* < .001. As expected, participants in the self-image threat condition also assessed that they had lower self-esteem (*M* = 3.24; *SD* = 0.64) than participants in the control condition (*M* = 4.24; *SD* = 0.66), *t*(72) = 9.75; *p* < .001. In addition we found that fear of invalidity and self-esteem were correlated (*r* = −.28, *p* = .014).

### Self-image threat and stereotyping: the moderating effect of NFC

We analysed the results exactly the same as in Study 1 and 2, using PROCESS macro (Hayes 2013, model 1 with heteroscedasticity-consistent standard errors estimator). The experimental conditions were coded (0 control/1 self-image threat), as predictor we used experimental condition, as a moderator NFC (mean centered), gender as a covariate and stereotype index as a dependent variable.

**Fig. 3:**
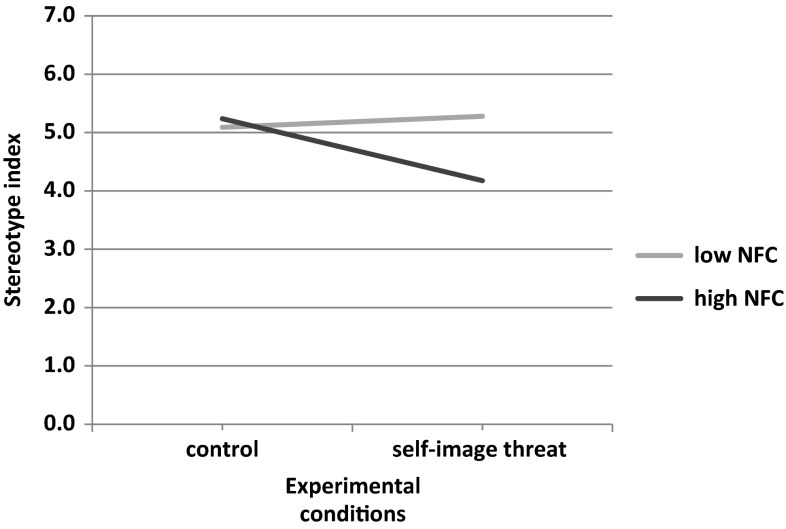
Regression lines showing stereotypical use of information as a function of self-image threat and NFC (Study 3)

Similar as in Study 1, there was a marginal effect of the self-image threat (vs. control) condition (*b* = −.44*; t*(69) = 1.83; *p* = .071; 95 % CI [−0.91, 0.04]), and no effect of gender (*b* = .17*; t*(69) = 0.70; *p* = .489; 95 % CI [−0.31, 0.65]). The only statistically significant effect was the interaction term between condition and NFC (*b* = −1.04*; t*(69) = 2.20; *p* = .031; 95 % CI [−1.98, −0.10]). The interaction pattern is depicted in Fig. 3.

As before, to probe for the significant interaction, we used a simple slope analysis and calculated the effect of our predictor (experimental condition) on DV at low (–1 SD) and high (+1 SD) values of the moderator (NFC). This analysis indicated that self-image threat was negatively related to the stereotype index at high NFC levels (*b* = −1.06; *t*(69) = 2.46*; p* = .016; 95 % CI [−1.92; −0.20] and was not related to it at low NFC levels (*b* = .19*; t*(69) = .63*; p* = .530; 95 % CI [−0.41; 0.78].

### Moderated mediation of self-image threat, fear of invalidity, and NFC on stereotypical perception

**Fig. 4:**
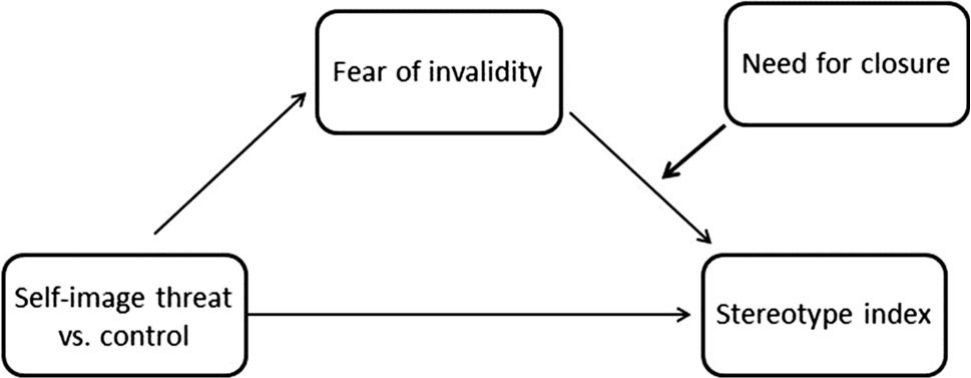
Moderated mediation model of the relationship between self-image threat, fear of invalidity, NFC and stereotyping

To test the effects of self-image threat, fear of invalidity, and NFC on stereotypical perception, we used the PROCESS macro (Hayes 2013; model 14 with heteroscedasticity-consistent standard errors estimator, and 10,000 bootstrapped samples for bias corrected confidence intervals). The model of the tested relationships is presented in Fig. 4. We expected that self-image threat (vs. control) predicts less stereotypical perception at high (vs. low) NFC levels in part because of the fear of invalidity. Thus, we expected that the indirect effect of self-image threat through fear of invalidity on stereotypical perceptions would appear only at high but not at low levels of NFC.

**Fig. 5:**
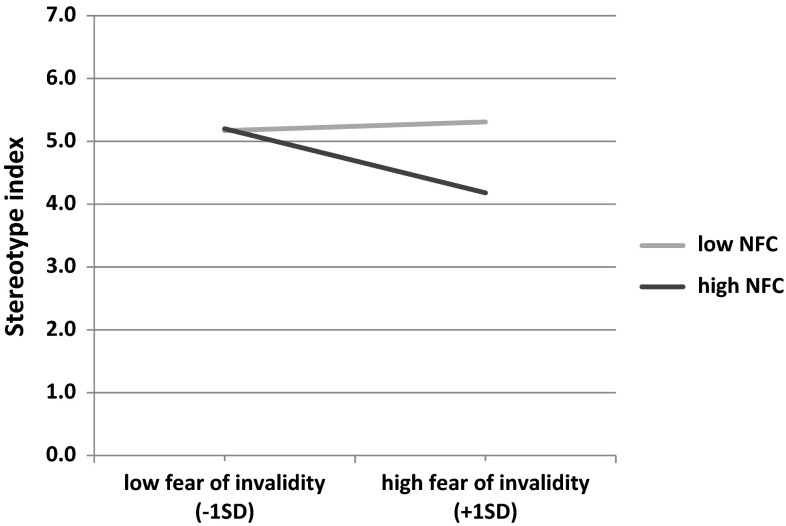
Regression lines showing stereotypical use of information as a function of fear of invalidity and NFC (Study 3)

Continuous variables except DV were mean-centered. We controlled for gender and self-esteem. With regard to stereotyping, the model included self-image threat, fear of invalidity, NFC, and the interaction between fear of invalidity and NFC. Direct effect of self-image threat on stereotype index was statistically non-significant (*b* = −.12; *t*(67) = .34; *p* = .734; 95 % CI [−0.83; 0.58]). However, indirect effect of self-image threat through fear of invalidity on stereotype index was negative and statistically significant only at high NFC levels (*b* = −.81; 95 % CI [−1.62; −0.17]). At low NFC levels, this effect was statistically non-significant (*b* = . 19; 95 % CI [−0.34; 0.79]). An index of moderated mediation was calculated (Hayes 2013) and it was significant (−.83, 95 % CI [−1.51; −0.26]). To visualize the interaction, we plotted the relationship between fear of invalidity and stereotype index at low (-1SD) and high (+1SD) values of NFC. This interaction is presented in Fig. 5. It shows that there is a negative relationship between fear of invalidity and stereotyping at high NFC levels (*b* = −.36, *t*(67) = 2.09, *p* = .040; 95 % CI [−0.70; −0.02]); this relationship was statistically non-significant at low NFC levels (*b* = .08, *t*(67) = .59, *p* = .554; 95 % CI [−0.19; 0.36]).

To determine whether decreased self-esteem is responsible for the effect of self-image threat on stereotyping, we repeated the above analysis with self-esteem as a mediator when controlling for the fear of invalidity and gender. We did not find a significant conditional indirect effect of self-image threat through self-esteem on the stereotype index (for low NFC: .07, 95 % CI [−0.31, 0.66], for high NFC: −.37, 95 % CI [−0.97, 0.07]), nor a significant interaction effect of NFC on the relationship between self-esteem and stereotyping (index of moderated mediation: −.37, 95 % CI [−1.09, 0.12]).

Thus, just like in Studies 1 and 2, self-image threat (vs. control) was negatively related to stereotypical perception of the outgroup at high but not low NFC levels. This results suggests that it is fear of invalidity—and not self-esteem—that yields less uniform and stereotypical perception of the outgroup among people high in NFC under self-image threat. This is an important result in light of previous findings showing the self-enhancing function of stereotyping and prejudice for perceivers, following a threat to their self-image (Fein and Spencer 1997). It also strengthens our argument regarding the role of fear of invalidity as both an outcome of self-image threat manipulation and as a mechanism mediating the self-image threat—stereotyping link.

## Meta-analysis

Given that each study only differed in terms of the materials that were used, and that other manipulations were not included, we report the integrated results using a meta-analysis of the three experiments (Cumming 2014). The meta-analysis was conducted using Comprehensive Meta-Analysis Software, on standardized regression coefficients (NFC as well as DV were standardized prior to analysis) and its standard errors. The analysis was performed on values of regression coefficients for the predictor (self-image threat vs. control) on a DV (stereotype index), at low (−1 SD) and high (+1 SD) values of NFC, obtained from simple slope analyses. We analyzed data from three studies, in two within-study subgroups (low vs. high NFC). We used the random-effects model, as it is appropriate and more realistic in this case (Schmidt et al. 2009).

**Fig. 6:**
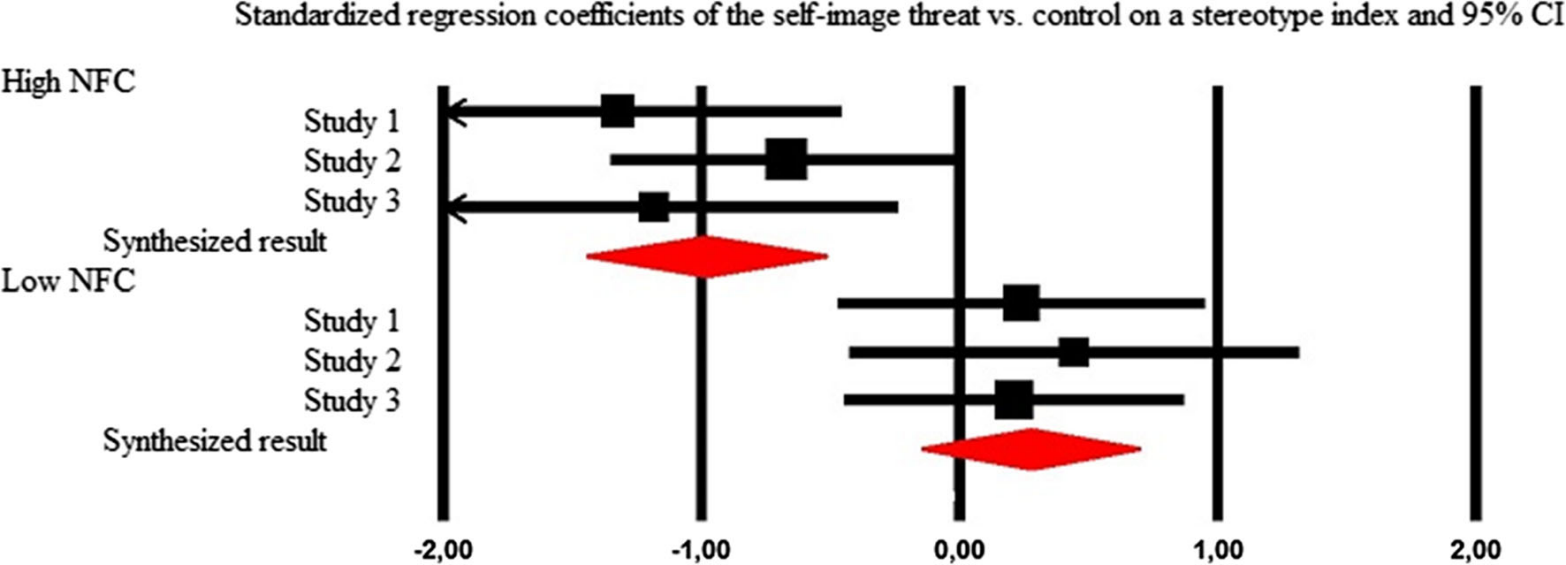
Meta-analysis of three current studies. Error bars represent 95 % confidence intervals

The calculated effect sizes and confidence intervals are reported in Fig. 6. The heterogeneity of effects sizes was not statistically significant (high NFC: *Q*(2) = 1.59, *p* = .451, *I* ^*2*^ = 0.00 %; low NFC: *Q*(2) = .19, *p* = .908, *I* ^*2*^ = 0.00 %). As predicted, the analysis indicated that self-image threat (vs. control) was negatively and statistically significantly related to stereotypical perception at high NFC levels (−0.98, *p* < .001, 95 % CI [−1.45, −0.52]) and positively but not statistically significantly at low NFC levels (.28, *p* = .198, 95 % CI [−0.14, 0.69]). Moreover, the difference between these two conditions was highly significant, as indicated by high between-group variance component *Q*(1) = 15.58, p < 0.001.

The results of this meta-analysis support our notion that self-image threat in domains of both competence and morality decreases stereotypical perception of outgroups at high but not low NFC levels.

## General discussion

Even though past research revealed that self-image threat may lead people to engage in stereotypical perception and prejudiced evaluations of others (e.g. Brown and Gallagher 1992; Gibbons and Gerrard 1991; Abrams and Hogg 1988), a growing body of evidence showed that it may not always be the case (e.g., Brockner and Chen 1996; Monin and Miller 2001; Petersen and Blank 2003; Sachdeva et al. 2009; Seta and Seta 1992). In the present research we focused on the moderating role of NFC, as it is important cognitive motive influencing social cognition. In a series of three studies we found that self-image threat predicts less stereotypical perception of the outgroups at high NFC levels. This relationship is not significant at low levels of NFC. In addition in Study 3 we demonstrated that fear of invalidity is responsible for these effects. We claimed that discrepancy between the need to maintain a positive self-image and threatening information about self, induces a fear of invalidity that undermines individuals’ confidence in their judgmental competence, thus lessening their reliance on habitual modes of judgment based on pre-existing knowledge structures and stereotypes. As we have seen, high NFC individuals stereotyped less than low NFC people in self–image threat/fear of invalidity condition. Built on motivational readiness theory (Kruglanski et al. 2014), we claimed that if *Want* × *Expectancy* defines the individual’s *Goal* to attain closure, fear of invalidity that increases in self-image threat condition, reduces the *Expectancy* of attaining the *Goal* similarly for people low and high in NFC. But the observed consequences of it are more pronounced for high than for low NFC people, because their *Want* to attain closure is greater, thus, decrease in *Expectancy* reduces the overall *Goal* strength relatively stronger for people high as compared to low in NFC. In consequence, people high in NFC who are exposed to self-threatening information are more sensitive to non-stereotypical information, attend to and examine more information about groups and ultimately develop more balanced perceptions of groups. Moreover, we demonstrated this effect when using different self-image threat manipulation and different targets, and thus different contents of stereotypes, which suggests that our present results reveal a general phenomenon, independent of self-image threat manipulation, group particularities and evaluations measures. A similar result was obtained by Rios et al. (2014), who showed that self-uncertainty leads to the need to highlight one’s distinctiveness and increases creative generation, thus unfreezing processes related to lower reliance on stereotypes.

Kosic et al. (2014) found, however, that self-image threat leads to more stereotyping among high (vs. low) NFC. We do not find this result contradictory to ours, as their effect was found only among highly prejudiced people, i.e., having generalized, strong and negative chronic beliefs about the outgroup. For these individuals, self-image threat may not undermine the confidence in their beliefs (what we see as important mechanism responsible for our findings). Thus it seems reasonable that prejudiced people are likely to cope with a self-image threat by direct negative evaluation of such groups. Moreover, in our research, we examined how participants under self-image threat modify their perceptions of stereotyped groups based on their NFC. In contrast, Kosic et al. (2014) manipulated participants’ motivation to express or not to express certain intergroup attitudes. We believe that both lines of research tap different mechanisms, whereas ours refers to social perception and knowledge creation processes among high NFC individuals under threat, Kosic and colleagues address the role of prejudice in the process of outgroup evaluation by high NFC people under self-image threat.

One might argue that our manipulation of self-image threat was confounded with self-esteem. In the self-image threat condition participants received explicit feedback that they had failed on a task, which may also influence their self-esteem. To address this concern, in Study 3 we measured self-esteem subsequent to the manipulation, so as to directly test this possibility. We demonstrated that although self-image threat manipulation did influence participants’ self-esteem, the latter is not responsible for self-image effects on stereotyping. This is in line with a meta-analysis by Aberson et al. (2000) indicating that self-esteem is not a good predictor of stereotyping and prejudiced evaluation.

We did not focus on the main effect of self-image threat but on joined effect of self-image and NFC on stereotypical perception of the outgroup, as recent findings showed that the relationship between self-image threat and stereotyping is moderated by other variables. For example, Florack et al. (2005) demonstrated that threat to an individual’s self-image leads to greater derogation of outgroup targets but only when the individual holds negative attitudes towards the respective outgroup (for similar results see Gagnon and Bourhis 1996). In contrast, when individuals have positive outgroup attitudes they do not perceive an outgroup target more negatively as a consequence of self-image threat. This is so because prejudiced responses are not a tool to affirm the self for people with positive or tolerant out-group attitudes. Self-affirmation theory (Steele et al. 1993) predicts that self-image distress may be reduced by expressing one’s values and attitudes; consequently, stereotypical and prejudiced responses should be an effective means of bolstering the self to the degree they are congruent with the individual’s beliefs about the outgroups or attitudes. But for individuals with positive outgroup attitudes, discrimination of an outgroup target should be inconsistent with their attitudes and, therefore, a further source for threat rather than self-affirmation. Similarly, other studies demonstrated that self-image threat leads to more stereotyping only among participants with reduced identification with the ingroup (Florack et al. 2005). These results are in line with predictions of social identity theory (Tajfel and Turner 1979) that individuals are more likely to derogate an outgroup member to bolster their self-esteem when they identify with their ingroup and hold positive attitudes toward it. Since for people with a negative attitude towards the in-group the latter is not an important part of the self, the comparison between ingroup and outgroup is irrelevant for their self-esteem and they should not discriminate the outgroup. Our research has shown that the strength of motivation toward closure can be another factor that moderates the impact of self-image threat on stereotyping. Therefore without consideration of moderators, there is no clear main effect of self-image threat on the evaluation of the outgroup target.

It is also noteworthy that we used explicit measures of stereotyping in our studies. Spencer and colleagues (Spencer et al. 1998) found evidence for stereotype activation after self-image threat (i.e., negative feedback) when perceivers were cognitively busy. It is possible that the self-image threat—outgroup stereotyping link would be more pronounced if implicit measures of stereotyping would be included, since they are less prone to be affected by response biases and social desirability. Further studies should address this possibility more in depth.

Although our results seems to be in contrast with some of literature on self-image threat effects (e.g., Fein and Spencer 1997), in fact they may be in line with previous findings. For example, some researchers suggest that a self-image threat increases the accessibility of negative and stereotypical information about out-group members to be used as a tool to restore self-esteem (e.g., Fein and Spencer 1997; Esses and Zanna 1995). Thus, when individuals are under a self-esteem threat, stereotypes of certain groups may more likely come to mind, and the threatened person interprets the other’s behavior in a stereotypical way and negative light. We suggest that high NFC individuals with high confidence to their beliefs about group (e.g., strong prejudice, strong beliefs that stereotype is correct) definitely should apply activated in self-image threat condition stereotype to the perception of the group (see Kosic et al. 2014). We, however demonstrated that when high NFC individuals are not confident in their beliefs (i.e., high fear of invalidity), they are more prone to stereotype less. Further, some authors suggest that a self-image threat induces a need to recover self-esteem, which, in turn, reduces motivation to inhibit stereotypes, negative attitudes and behaviors toward out-group members (Sinclair and Kunda 1999). We suggest that high NFC individuals who have strong beliefs about groups are probably also less prone to inhibit stereotypes, but if the confidence in their knowledge is not so strong or just undermined (as in our studies), they should be more prone to inhibit stereotypes more strongly (as they did in our studies). Finally, it is possible that self-image threat might induce a kind of ego-depletion mechanism (Baumeister 1998), so that threatened people do not have the cognitive energy needed to inhibit stereotype expressions. Again, there are some results showing that high in NFC people with unconstrained cognitive resources may engage in effortful processing in order to achieve their goal (Strojny et al. 2016), thus may also inhibit stereotype expression efficiently.

Finally, it is worth stressing that our study also contributes to NFC theory, showing that whereas early studies demonstrated that closure seeking individuals opt for easy and simplistic strategies of information processing, thus stereotyping, our research revealed that there are circumstances (i.e., self-image threat) in which individuals high in NFC engage in effortful and open-minded processing of information instead of relying on simplistic processing styles (see Roets et al. 2015).

We did not obtain a positive relationship between NFC and stereotyping in the control condition. In fact we performed an additional meta-analysis on the present three studies (with NFC as a predictor and self-image threat as a moderator) that demonstrated that in control condition NFC was positively although non-significantly related to stereotypical evaluations (0.13, *p* = .073, 95 % CI [−0.01, 0.28]). Possible reason explaining these results may be our use of explicit and direct evaluations measures. High NFC people are more sensitive to normative pressures (e.g., Fu et al. 2007; Jia et al. 2014) and might be motivated to not express their stereotypes or prejudices at an explicit level. In fact recently many studies demonstrated the effect of NFC on stereotyping only under certain circumstances (e.g., Kossowska et al. 2015; Kossowska and Bar-Tal 2013; Sun et al. 2016).

In sum, the present research shows that the impact of self-image threat on the judgment of outgroup members depends on individual differences in an important epistemic motivation, i.e., NFC. The stereotypical perception of an outgroup target is not inevitable. Rather, as presently demonstrated individuals may use different strategic responses in coping with self-image threat. The less stereotypical perception of a target may be one such response when NFC is high. Thus high but not low NFC people become sensitive to non-stereotypical information about the outgroup when forming impression. We believe that the proposed framework may be especially useful in explaining the variability in people`s responses to uncertain situations, especially when uncertainty refers to the self.

## Footnotes

1 We performed power analysis using G*Power software (Faul et al. 2009). Required sample size for testing for an increase in explained variance due to the interaction (which was our effect of interest), assuming power at level .80, *α* = .05, in the model with four predictors and a medium effect size *f*² = .15, was 55 participants. Although we did not reach that amount in Study 1, we believe that we at least partially addressed the problem of the lack of power at .80 in this single study by performing a meta-analysis, which allow for a more precise estimation of the population effect size (e.g., Braver et al. 2014; Cumming 2014).

2 The Decisiveness facet scale has been particularly criticized because it measures a distinct dimension (see Neuberg et al. 1997; Kossowska et al. 2002) and because its construct validity is doubtful (see Roets et al. 2006). Hence, it was discussed to what extent Decisiveness played its role as one of the five subscales contributing to the global need for closure score or whether it is related to a different underlying process and refers to a distinctive need for closure factor (Kossowska 2007). Roets and Van Hiel (2007) demonstrated that the traditional Decisiveness scale taps a mixture of the hypothesized “need” construct as well as unintended ability content (see also Bar-Tal and Kossowska 2010).

3 In original procedure by Fein and Spencer (1997) “the experimenter explained that they had been assigned to a control condition in which they were simply to read the materials contained in a bogus test of intelligence” (p. 35). We tested this procedure before and turned out that participants knowing that they need to just read and not solve the IQ test, were completely unmotivated to do that—thus for sake for ecological validity of the procedure we asked them to solve the task informing about points they get but without information whether it is good or bad result. That might create the situation that some of them evaluated their performance according to their subjective standards.

4 Participants answered also five more questions from the scale of Bar-Tal and Kossowska (2010) measured ability to achieve closure. As irrelevant to the study, the construct and the items were not mentioned in the paper. We however repeated all the analysis including these items (as ability index) and they did not change the results. Thus we reported analysis without them to make the story comprehensive.

## References

[CR1] Aberson CL, Healy M, Romero V (2000). Ingroup bias and self-esteem: A meta-analysis. Personality and Social Psychology Review.

[CR2] Abrams D, Hogg M (1988). Comments on the motivational status of self-esteem in social identity and intergroup discrimination. European Journal of Social Psychology.

[CR3] Ammons RB, Ammons CH (1962). The quick test: Provisional manual. Psychological Reports.

[CR4] Atkinson JW (1964). An introduction to motivation.

[CR5] Bar-Tal Y, Kossowska M, Villanueva JP (2010). The efficacy at fulfilling need for closure: The concept and its measurement. Personality traits: Classification, effects and changes.

[CR6] Baumeister RF, Gilbert DT, Fiske ST, Lindzey G (1998). The self. Handbook of social psychology.

[CR7] Bliss-Moreau E, Barrett LF, Wright CI (2008). Individual differences in learning the affective value of others under minimal conditions. Emotion.

[CR8] Braver SL, Thoemmes FJ, Rosenthal R (2014). Continuously cumulating meta-analysis and replicability. Perspectives on Psychological Science.

[CR9] Brockner J, Chen Y-R (1996). The moderating roles of self-esteem and self-construal in reaction to a threat to the self: Evidence from the People’s Republic of China and the United States. Journal of Personality and Social Psychology.

[CR10] Brown J, Gallagher F (1992). Coming to terms with failure: Private self-enhancement and public self-effacement. Journal of Experimental Social Psychology.

[CR11] Cichocka A, Winiewski M, Bilewicz M, Bukowski M, Jost J (2015). Complementary stereotyping of ethnic minorities predicts system justification in Poland. Group Processes & Intergroup Relations.

[CR12] Cosmides L (1989). The logic of social exchange: Has natural selection shaped how humans reason? Studies with the Wason selection task. Cognition.

[CR13] Cottrell CA, Neuberg SL, Li NP (2007). What do people desire in others? A sociofunctional perspective on the importance of different valued characteristics. Journal of Personality and Social Psychology.

[CR14] Crocker J, Thompson LL, McGraw KM, Ingerman C (1987). Downward comparison, prejudice, and evaluation of others: Effects of self-esteem and threat. Journal of Personality and Social Psychology.

[CR15] Cuddy A, Fiske S, Glick P (2008). Warmth and competence as universal dimensions of social perception: The stereotype content model and the BIAS map. Advances in Experimental Social Psychology.

[CR16] Cumming G (2014). The new statistics: Why and how. Psychological Science.

[CR17] Driscoll DM, Hamilton DL, Sorrentino RM (1991). Uncertainty orientation and recall of person-descriptive information. Personality and Social Psychology Bulletin.

[CR18] Ehrlich HJ (1973). The social psychology of prejudice.

[CR19] Esses VM, Zanna MP (1995). Mood and the expression of ethnic stereotypes. Journal of Personality and Social Psychology.

[CR20] Faul F, Erdfelder E, Buchner A, Lang AG (2009). Statistical power analyses using G* Power 3.1: Tests for correlation and regression analyses. Behavior Research Methods.

[CR21] Fein S, Spencer SJ (1997). Prejudice as self-image maintenance: Affirming the self through derogating others. Journal of Personality and Social Psychology.

[CR22] Florack A, Scarabis M, Gosejohann S (2005). The effects of self-image threat on the judgment of out-group targets. Swiss Journal of Psychology/Schweizerische Zeitschrift Für Psychologie/Revue Suisse de Psychologie.

[CR23] Freund T, Kruglanski AW, Shpitzajzen A (1985). The freezing and unfreezing of impressional primacy: Effects of the need for structure and the fear of invalidity. Personality and Social Psychology Bulletin.

[CR24] Fu JH, Morris MW, Lee S, Chao M, Chiu C, Hong Y (2007). Epistemic motives and cultural conformity: Need for closure, culture, and context as determinants of conflict judgments. Journal of Personality and Social Psychology.

[CR25] Gagnon A, Bourhis RY (1996). Discrimination in the minimal group paradigm: Social identity theory or self-interest?. Personality and Social Psychology Bulletin.

[CR26] Gibbons F, Gerrard M, Suls J, Wills T (1991). Downward comparison and coping with threat. Social comparison: Contemporary theory and research.

[CR27] Guinote A, Brown M, Fiske ST (2006). Minority status decreases sense of control and increases interpretive processing. Social Cognition.

[CR28] Hayes A (2013). Introduction to mediation, moderation, and conditional process analysis.

[CR29] Hayes AF, Cai L (2007). Using heteroskedasticity-consistent standard error estimators in OLS regression: An introduction and software implementation. Behavior Research Methods.

[CR30] Heatherton TF, Polivy J (1991). Development and validation of a scale for measuring state self-esteem. Journal of Personality and Social Psychology.

[CR31] Hull CL (1943). Principles of behavior: An introduction to behavior theory.

[CR32] Jia L, Hirt ER, Evans DN (2014). Putting the freeze on priming: The role of need for cognitive closure on the prime-norm dynamic. Personality and Social Psychology Bulletin.

[CR33] Kofta M, Narkiewicz-Jodko W (2003). Poziom uprzedzen, deprywacja kontroli a stereotypowe przetwarzanie informacji na temat Cyganów [Prejudice, control deprivation and stereotypical information processing about Gypsies]. Studia Psychologiczne.

[CR34] Kofta M, Sędek G (2005). Conspiracy stereotypes of Jews during systemic transformation in Poland. International Journal of Sociology.

[CR35] Kosic A, Mannetti L, Livi S (2014). Forming impressions of in-group and out-group members under self-esteem threat: The moderating role of the need for cognitive closure and prejudice. International Journal of Intercultural Relations.

[CR36] Kossowska M (2007). Motivation towards closure and cognitive processes: An individual differences approach. Personality and Individual Differences.

[CR37] Kossowska M, Bar-Tal Y (2013). Need for closure and heuristic information processing: The moderating role of the ability to achieve the need for closure. British Journal of Psychology.

[CR38] Kossowska M, Dragon P, Bukowski M (2015). When need for closure leads to positive attitudes towards a negatively stereotyped outgroup. Motivation and Emotion.

[CR39] Kossowska M, Van Hiel A, Chun WY, Kruglanski AW (2002). The Need for Cognitive Closure scale: structure, cross-cultural invariance, and comparison of mean ratings between European-American and East Asian samples. Psychologica Belgica.

[CR40] Kruglanski AW (1989). The psychology of being ‘‘right’’: The problem of accuracy in social perception and cognition. Psychological Bulletin.

[CR41] Kruglanski AW, Bélanger JJ, Chen X, Köpetz C, Pierro A, Mannetti L (2012). The energetics of motivated cognition: A force-field analysis. Psychological Review.

[CR42] Kruglanski AW, Chernikova M, Rosenzweig E, Kopetz C (2014). On motivational readiness. Psychological Review.

[CR44] Kruglanski AW, Shah JY, Pierro A, Mannetti L (2002). When similarity breeds content: Need for closure and the allure of homogeneous and self-resembling groups. Journal of Personality and Social Psychology.

[CR45] Lewin K (1935). A dynamic theory of personality.

[CR46] Monin M, Miller D (2001). Moral credentials and the expression of prejudice. Journal of Personality and Social Psychology.

[CR47] Neuberg SL, Judice TN, West SG (1997). What the Need for Closure Scale measures and what it does not: Toward differentiating among related epistemic motives. Journal of Personality and Social Psychology.

[CR48] Petersen LE, Blank H (2003). Ingroup bias in the minimal group paradigm shown by three-person groups with high or low state self-esteem. European Journal of Social Psychology.

[CR49] Rios K, Markman KD, Schroeder JR, Dyczewski E (2014). A (creative) portrait of the uncertain individual: Self-uncertainty and individualism enhance creative generation. Personality and Social Psychology Bulletin.

[CR50] Roets A, Kruglanski AW, Kossowska M, Pierro A, Hong Y, Olson James, Zanna Mark (2015). The motivated gatekeeper of our minds: New directions in need for closure theory and research. Advances in experimental social psychology.

[CR51] Roets A, Van Hiel A (2007). Separating ability from need: clarifying the dimensional structure of the Need for Closure Scale. Personality and Social Psychology Bulletin.

[CR52] Roets A, Van Hiel A, Cornelis I (2006). The dimensional structure of the need for cognitive closure scale: relationships with “seizing” and “freezing” processes. Social Cognition.

[CR53] Sachdeva S, Iliev R, Medin D (2009). Sinning saints and saintly sinners: The paradox of moral self-regulation. Psychological Science.

[CR54] Schmidt FL, In-Sue Oh, Hayes TL (2009). Fixed-versus random-effects models in meta-analysis: Model properties and an empirical comparison of differences in results. British Journal of Mathematical and Statistical Psychology.

[CR55] Seta CE, Seta JJ (1992). Observers and participants in an intergroup setting. Journal of Personality and Social Psychology.

[CR56] Shah JY, Kruglanski AW, Thompson EP (1998). Membership has its (epistemic) rewards: Need for closure effects on in-group bias. Journal of Personality and Social Psychology.

[CR57] Sinclair L, Kunda Z (1999). Reactions to a lack professional: Motivated inhibition and activation of conflicting stereotypes. Journal of Personality and Social Psychology.

[CR58] Spence KW (1937). The differential response in animals to stimuli varying within a single dimension. Psychological Review.

[CR59] Spencer SJ, Fein S, Wolfe CT, Fong C, Dunn MA (1998). Automatic activation of stereotypes: The role of self-image threat. Personality and Social Psychology Bulletin.

[CR60] Steele CM, Spencer SJ, Lynch M (1993). Self-image resilience and dissonance: The role of affirmational resources. Journal of Personality and Social Psychology.

[CR61] Stephan WG, Rosenfield D (1978). Effects of desegregation on racial attitudes. Journal of Personality and Social Psychology.

[CR62] Strojny P, Kossowska M, Strojny A (2016). Search for expectancy-inconsistent information reduces uncertainty better: The role of cognitive capacity. Frontiers in Psychology.

[CR63] Sun S, Zuo B, Wu Y, Wen F (2016). Does perspective taking increase or decrease stereotyping? The role of need for cognitive closure. Personality and Individual Differences.

[CR64] Tajfel H, Turner JC, Austin WG, Worchel S (1979). An integrative theory of intergroup conflict. The social psychology of intergroup relations.

[CR65] Thompson MM, Naccarato ME, Parker KE (1986). Personal Fear of Invalidity Scale. PsycTESTS.

[CR66] Thompson MM, Naccarato ME, Parker KCH, Moskowitz GB, Moskowitz GB (2001). The personal need for structure and personal fear of invalidity measures: Historical perspectives, current applications, and future directions. Cognitive social psychology: The Princeton symposium on the legacy and future of social cognition.

[CR67] Tolman EC (1955). Principles of performance. Psychological Review.

[CR68] Webster D, Kruglanski A (1994). Individual differences in need for cognitive closure. Journal of Personality and Social Psychology.

[CR69] Węglarczyk, A. (2001). Grzech czy oznaka życiowej zaradności [Sin or the sign of life resourcefulness]. *Forum Akademickie*, *3*, http://forumakad.pl/archiwum/2001/03/artykuly/12-grzech_czy_oznaka_zaradnosci.htm

[CR70] Wojciszke B (2005). Morality and competence in person- and self-perception. European Review of Social Psychology.

